# Ornithine Transcarbamylase Deficiency: If at First You Do Not Diagnose, Try and Try Again

**DOI:** 10.1155/2017/8724810

**Published:** 2017-11-27

**Authors:** Christan D. Santos, Robert A. Ratzlaff, Jennifer C. Meder, Paldeep S. Atwal, Nicole E. Joyce

**Affiliations:** ^1^Department of Critical Care Medicine, Mayo Clinic, Jacksonville, FL, USA; ^2^Department of Nutritional Services, Mayo Clinic, Jacksonville, FL, USA; ^3^Department of Clinical Genomics, Mayo Clinic, Jacksonville, FL, USA; ^4^Division of Hospital Internal Medicine, Mayo Clinic, Jacksonville, FL, USA

## Abstract

Ornithine transcarbamylase (OTC) deficiency is well known for its diagnosis in the neonatal period. Presentation often occurs after protein feeding and manifests as poor oral intake, vomiting, lethargy progressing to seizure, respiratory difficulty, and eventually coma. Presentation at adulthood is rare (and likely underdiagnosed); however, OTC deficiency can be life-threatening and requires prompt investigation and treatment. Reports and guidelines are scarce due to its rarity. Here, we present a 59-year-old woman with a past history of irritable bowel syndrome who underwent a reparative operation for rectal prolapse and enterocele. Her postoperative course was complicated by a bowel perforation (which was repaired), prolonged mechanical ventilation, tracheostomy, critical illness myopathy, protein-caloric malnutrition, and altered mental status. After standard therapy for delirium failed, further investigation showed hyperammonemia and increased urine orotic acid, ultimately leading to the diagnosis of OTC deficiency. This case highlights the importance of considering OTC deficiency in hospitalized adults, especially during the diagnostic evaluation for altered mental status.

## 1. Introduction 

Ornithine transcarbamylase (OTC) deficiency (also referred to as ornithine carbamoyltransferase deficiency) is an X-linked proximal urea cycle disorder that results in a spectrum of severe neonatal-onset disease in boys, rarely in girls, and a milder adult-onset presentation. In the urea cycle, OTC combines ornithine and carbamoyl phosphate to form citrulline. Thus, an OTC enzyme deficiency leads to decreased production of citrulline, interruption of the urea cycle, increased orotic acid which is produced by excess carbamoyl phosphate, and ultimately life-threatening hyperammonemia due to the inability to excrete excess nitrogen. In females, the severity of the condition depends on the amount of X-inactivation of the pathogenic OTC allele and resultant OTC enzyme activity in the liver [1].

OTC deficiency can range from asymptomatic to severely symptomatic in heterozygous adults; those with a milder disease course often report a history of self-limiting protein intake. Acute hyperammonemic episodes can occur and are often triggered by major illness, surgery, pregnancy, corticosteroid and valproic acid administration, prolonged fasting, increased protein intake, and total parenteral nutrition (TPN) [2–6].

These hyperammonemic episodes present with neurologic abnormalities such as encephalopathy, psychosis, and delirium, which can progress to coma and death. Although limited, some reports describe the severity of this disease and emphasize the risk of death if undiagnosed. Brassier et al. [1] reviewed the long-term outcomes of 90 patients with OTC deficiency: 70% presented after the neonatal period and 9% of the late onset group (>1 month of age) died on initial presentation. Batshaw et al. [7] analyzed a larger group of patients (*n* = 614), and 66% of patients presented after the neonatal period; the overall mortality rate in the late onset age group was 11%.

## 2. Case Presentation

A 59-year-old woman with a history of irritable bowel syndrome and protein aversion, which led to a preferential vegetarian diet, was admitted for elective reparative surgery to treat rectal prolapse and enterocele. Her postoperative course was complicated by bowel perforation (requiring small bowel resection and end ileostomy), fecal peritonitis, intra-abdominal abscesses, septic shock, respiratory failure, tracheostomy, critical illness myopathy, protein-caloric malnutrition, and altered mental status (AMS). The patient provided written informed consent to publish these findings.

The patient's daily spontaneous awakening trials during her intensive care unit (ICU) course showed a positive evaluation on the Confusion Assessment Method for the ICU, score of +3 on the Richmond Agitation and Sedation Scale, and inability to follow 2-step commands, which led to an initial diagnosis of delirium. After standard delirium treatment was unsuccessful, further ICU investigation yielded normal renal, thyroid, and liver function. However, she had a markedly elevated ammonia level (100 mcmol/L, normal 0–30). Noncontrast head computed tomography and magnetic resonance imaging of the brain were negative for acute intracranial process.

Electroencephalography showed no evidence of nonconvulsive status epilepticus. Urine orotic acid and plasma amino acid panel were ordered to investigate her unexplained hyperammonemia. Her urine orotic acid level was elevated (3.2 mmol/mol creatinine, normal 0.4–1.2) but the plasma glutamine (normal 371–957 nmol/ml), ornithine (normal 38–130 nmol/ml), citrulline (normal 17–46 nmol/ml), arginine (normal 30–120 nmol/ml), and argininosuccinic acid (normal <2 nmol/ml) were within normal ranges. Nitrogen scavengers were not initiated at this time since there were no abnormalities in the rest of the urea cycle panel, ammonia began to trend down, and her mental status subsequently improved.

Because of prolonged mechanical ventilation and weaning failure, the patient received a standard polymeric enteral formula with a modular protein supplement. This was transitioned to an isotonic enteral formula after her ileostomy output increased. Her enteral nutrition was inconsistent during her ICU stay because of intestinal perforation, multiple planned procedures, intermittent high doses of vasopressors, and fluctuating doses of propofol.

On day 25 in the hospital, she was transferred to the step-down unit at her baseline mental state. Her appetite remained poor as she continued to experience bloating, nausea, and high gastric residual volume resulting in intermittent enteral and oral feeding. Trials of antiemetics, appetite stimulants, nutrition supplements, and diet adjustments were unsuccessful.

In the days approaching discharge, she had acute neurologic changes overnight, including visual hallucinations, hyperreflexia, clonus, generalized high-frequency low-amplitude tremors, nystagmus, and delirium. During the same night, she received the longest period of continuous enteral feeding since admission.

After this sudden neurologic decline on the step-down unit, OTC deficiency as a diagnosis was investigated further and empiric treatment was immediately initiated. Urine organic acid and acylglycines, quantitative plasma amino acid, and quantitative plasma acylcarnitine panels were ordered to screen for inborn errors of metabolism. This additional testing confirmed increased orotic acid, low citrulline, and elevated ammonia levels. The constellation of elevated urine orotic acid, low citrulline, persistent hyperammonemia with normal liver function, ketonuria, feeding difficulty, and neurologic changes were so highly suspicious of OTC deficiency; a confirmation of diagnosis by means of liver biopsy was not performed.

## 3. Treatment

This patient's management was based on consultation with a biochemical geneticist and focused on 4 main areas: restriction of protein, promotion of anabolism, administration of nitrogen-scavenging medications, and monitoring. Protein intake was withheld for 24 hours at treatment onset and then limited to 0.5 g/kg per day with a calorie goal of 30 kcal/kg. In this case, protein restriction could not be achieved through the available enteral formulas in the inpatient setting. TPN was initiated with dextrose (100 g), a lipid emulsion (28 g), and standard essential amino acids (25 g). Rohr recommends 30% to 50% of total protein be given as essential amino acids, a provision that is important for preventing chronic protein insufficiency [8].

To stimulate the urea cycle, intravenous arginine was initiated at a continuous rate of 600 mg/kg per day, along with 10% dextrose in normal saline at 125 cc/hr [6]. A glucose goal of 100 to 150 mg/dL was maintained with an insulin drip.

Ammonia scavenger therapy with intravenous sodium benzoate and sodium phenylacetate was initiated. Hemodialysis can be used for refractory hyperammonemia, but this intervention was not needed by our patient [6]. While receiving these interventions, her serum sodium and ammonia levels were monitored every 4 hours and her serum amino acid levels were monitored every 48 hours.

This course was continued until she became neurologically stable, at which time her oral discharge regimen was initiated. This regimen included a specially ordered, protein-free infant formula mixed with almond milk. She was instructed to consume 3 portions of this supplement daily, with a protein intake goal of less than 25 g per day. She received nutritional education regarding the effects of protein in patients with OTC deficiency and the importance of protein restriction. She was discharged with oral arginine (500 mg, 3 times per day). On follow-up, she was transitioned to oral citrulline (1.3 g, 3 times per day) (3 g/m^2^) and sodium phenylbutyrate (4.5 g, 3 times per day) (10 g/m^2^).

## 4. Outcome and Follow-Up

After prompt initiation of OTC specific treatment including nitrogen scavengers and restricted protein, her neurologic symptoms improved over 48 hours, and by 72 hours, she had returned to her baseline mental and neurologic state. Her inpatient oral intake adequately met goals, and TPN was discontinued. She was transitioned to a short-term rehabilitation facility, and on discharge to home, she was eating a regular-texture oral diet and supplementing with low-protein formula. Unfortunately, this diet was not sustained over several weeks, and she had failure to thrive. She was readmitted to the hospital for reinitiation of TPN in addition to oral diet. Upon final discharge, outpatient follow-up included consultations with a geneticist and genetic dietician.

## 5. Discussion

This case depicts a unique diagnosis (OTC) to a common clinical dilemma (AMS). The most common causes of AMS are cerebrovascular, traumatic, metabolic, infectious, psychiatric, and endocrine in origin [9].  Table 1 includes the broad range of disorders that can result in AMS.

OTC deficiency is not routinely considered during an investigation of AMS; however, early diagnosis and treatment are imperative to prevent severe neurologic injury and death [6]. For our patient, OTC deficiency was considered in the differential diagnosis because of her persistent hyperammonemia and nutritional history (preferential vegetarian diet) and the consistent failure of traditional delirium therapies. This case exemplifies the important role of multidisciplinary collaboration and communication in managing this uncommon deficiency in a urea cycle enzyme. During this patient's course, consultants from the departments of intensive care, neurology, internal medicine, nutrition, and genetics all had pivotal roles in diagnosing and treating this patient's disease.

## Figures and Tables

**Table 1:** Differential diagnosis of altered mental status.

Origin of AMS	Differential diagnosis
Infection	Urinary tract infection, pneumonia, bacteremia, meningitis, encephalitis, brain abscess, neurosyphilis, fever
Withdrawal	Alcohol, sedative-hypnotic drugs, barbiturates, benzodiazepines
Acute metabolic condition	Acid-base disturbance, electrolyte imbalance, liver or renal failure
Trauma	Head injury, surgery, burns, heat stroke, hypothermia
Central nervous system pathology	Subdural hematoma, epidural hematoma, subarachnoid hemorrhage, hydrocephalus, seizure, stroke, tumor, demyelinating lesion, transient ischemic attack
Hypoxia or cardiopulmonary condition	Hypoxemia, hypotension or shock, arrhythmia, anemia, carbon monoxide poisoning, methemoglobinemia
Vitamin deficiency	Thiamine (Wernicke encephalopathy), niacin, vitamin B12
Endocrinopathy	Hyperadrenocorticism, hypoadrenocorticism, hyperglycemia, hypoglycemia, hyperthyroidism, hypothyroidism, hypercalcemia
Acute vascular condition	Hypertensive encephalopathy, stroke, arrhythmia, shock, thrombotic thrombocytopenic purpura, mesenteric ischemia
Toxin or drug toxicity	Sedatives, narcotics, anticholinergics, antipsychotics, neuroleptic malignant syndrome, serotonin syndrome, illicit drugs
Heavy metal poisoning	Lead, arsenic, manganese, mercury, thallium

## Abbreviations

AMS: Altered mental status
ICU: Intensive care unit
OTC: Ornithine transcarbamylase
TPN: Total parenteral nutrition.
